# Exosomes transmit T790M mutation‐induced resistance in EGFR‐mutant NSCLC by activating PI3K/AKT signalling pathway

**DOI:** 10.1111/jcmm.14838

**Published:** 2020-01-02

**Authors:** Xiaozhen Liu, Tao Jiang, Xuefei Li, Chao Zhao, Jiayu Li, Fei Zhou, Limin Zhang, Sha Zhao, Yijun Jia, Jinpeng Shi, Guanghui Gao, Wei Li, Jing Zhao, Xiaoxia Chen, Chunxia Su, Shengxiang Ren, Caicun Zhou

**Affiliations:** ^1^ Department of Medical Oncology Shanghai Pulmonary Hospital & Thoracic Cancer Institute Tongji University School of Medicine Shanghai China; ^2^ Department of Lung Cancer and Immunology Shanghai Pulmonary Hospital Tongji University School of Medicine Shanghai China

**Keywords:** EGFR‐TKI resistance, exosomes, NSCLC

## Abstract

Emerging evidence has shown that exosomes derived from drug‐resistant tumour cells are able to horizontally transmit drug‐resistant phenotype to sensitive cells. However, whether exosomes shed by EGFR T790M‐mutant–resistant NSCLC cells could transfer drug resistance to sensitive cells has not been investigated. We isolated exosomes from the conditioned medium (CM) of T790M‐mutant NSCLC cell line H1975 and sensitive cell line PC9. The role and mechanism of exosomes in regulating gefitinib resistance was investigated both in vitro and in vivo. Exosome‐derived miRNA expression profiles from PC9 and H1975 were analysed by small RNA sequencing and confirmed by qRT‐PCR. We found that exosomes shed by H1975 could transfer gefitinib resistance to PC9 both in vitro and in vivo through activating PI3K/AKT signalling pathway. Small RNA sequencing and RT‐PCR confirmed that miR‐3648 and miR‐522‐3p were the two most differentially expressed miRNAs and functional study showed that up‐regulation of miR‐522‐3p could induce gefitinib resistance in PC9 cell. The findings of our study reveal an important mechanism of acquired resistance to EGFR‐TKIs in NSCLC.

## 1 INTRODUCTION

With the tremendous development in understanding lung cancer biology, the treatment strategies for advanced lung cancer, especially non–small‐cell lung cancer (NSCLC), have changed and currently are based on genetic abnormalities.^1^, ^2^, ^3^ Although tremendous initial response to epidermal growth factor receptor tyrosine kinase inhibitors (EGFR‐TKIs) in EGFR‐mutant advanced NSCLC patients, acquired resistance inevitably develops and T790M mutation accounts for 50%‐60% resistant cases.^4^, ^5^, ^6^ Researchers have reported that genetic tolerance might arise from small group of drug‐resistant cells in patients.^7^ These resistant clones can continue to share molecular characteristics of the drug‐resistant cells, contributing to a attenuated apoptotic response to subsequent therapies.^7^ Therefore, the intratumoral heterogeneous of NSCLC becomes more complicated after EGFR‐TKI therapy. Although the mechanisms of acquired resistance of *EGFR*‐mutant NSCLC to EGFR‐TKIs have been identified,^4^ little is known about the transfer mechanism between EGFR‐TKI–resistant cells and sensitive cells and how this transmission affect drug response needs to be further elucidated.

Exosomes are membranous vesicles 30‐150 nm in diameter constitutively released by almost all cell types and play important roles in intercellular communication by transferring intercellular cargoes, such as miRNAs, mRNAs and proteins.^8^, ^9^, ^10^ As mediators between cells, emerging evidences have now indicated that exosomes derived from stromal cells can potentially affect therapeutic response and induce drug resistance of cancer cells.^11^, ^12^ Moreover, recently several researches have described that exosomes shed by drug‐resistant tumour cells can transmit drug‐resistant phenotype to drug‐sensitive cells, primarily by conferring drug‐efflux pumps and miRNAs.^13^, ^14^, ^15^ This has been recognized as a novel mechanism for the “dissemination” of drug resistance. Given the heterogeneous nature of NSCLC, whether EGFR‐TKI–resistant cell‐derived exosomes could promote drug resistance in sensitive cells need to be discussed.

MicroRNAs (miRNAs) are small, non‐coding RNAs. By directly binding to the 3′ untranslated regions (UTRs) of target mRNAs, they can deregulate or inhibit protein expression and regulate tumorigenesis, metastization and affect drug responses.^16^, ^17^ Interestingly, miRNAs packaged into exosomes can be protected against degradation by RNases, compared with naked miRNAs.^18^ Moreover, researches about the transfer of miRNAs by exosomes provided reliable evidence to demonstrate the study of using miRNAs carried by exosomes as markers of drug resistance.^13^, ^15^ However, few studies were carried out about the roles of miRNAs transmitted by exosomes in inducing EGFR‐TKI resistance in NSCLC.

Therefore, in our study, we investigated the contributions of exosomes shed by EGFR‐TKI–resistant NSCLC cells carrying *T790M* mutation to transferring drug resistance to sensitive cells and explored the potential mechanisms. Our work provides new insights into how tumour heterogeneous promotes drug resistance in acquired EGFR‐TKI resistance.

## 2 MATERIALS AND METHODS

### 2.1 Cell lines and cell culture

The NSCLC cell lines PC9 (EGFR exon 19 deletion) and H1975 (L858R/T790M) were cultured in DMEM (HyClone) supplemented with 10% fetal bovine serum (FBS) (Life Technologies) and 1% Penicillin Streptomycin (PS) (Life Technologies). All cells were incubated at 37°C in humidified air with 5% CO_2_.

### 2.2 Exosome experiments

After cells reached 80%‐90% confluency, we washed cells with phosphate‐buffered saline (PBS) (HyClone) for 3 times and incubated without FBS for 48 hours. Culture medium were collected and centrifuged at 2000 *g* for 30 minutes, followed by incubation with Total Exosome Isolation Kit (Life Technologies) at 4°C overnight. Exosomes were then harvested by centrifugation at 10 000 *g* for 60 minutes and resuspended in PBS. The concentration of exosomal proteins was quantified using a BCA protein assay kit (Beyotime Biotechnology). CD63 and GM130 (antibody for CD63 was obtained from Life Technologies, antibody for GM130 was purchased from abcam) expressions were measured using Western blot analysis. For in vitro exosome treatment, 100 μg (equivalent to those collected from 1 × 10^7^ producer cells) were added to 1 × 10^5^ recipient cells.

### 2.3 Transmission electron microscopy (TEM) and nanoparticle tracking analysis (NTA)

Isolated exosome samples were resuspended with PBS. About 10‐20 μL sample was dropped on the carbon grid for 1 minute. The droplet was sucked off with filter paper and contrasted with 2% uranyl acetate. Images were obtained with TEM (FEI Tecnai G2 spirit). The particle size and concentration of exosomes were measured by nanoparticle tracking analysis (NTA) using ZetaView PMX 110 (Particle Metrix) and corresponding software ZetaView 8.04.02. NTA measurements were recorded and analysed at 11 locations. The ZetaView system was calibrated using 100 nm polystyrene particles. Temperature was maintained around 23°C and 37°C.

### 2.4 Fluorescence microscopy analysis of exosome internalization

PC9 or H1975 cells were incubated with medium containing 5 μmol/L DiI (red) (Beyotime Biotechnology) at 37°C for 20 minutes and washed with PBS 3 times. We added DiO (Beyotime Biotechnology) into 100 μg exosome suspension at 5 μmol/L and incubated for 20 minutes, then washed by Exosome Spin Columns (Invitrogen) to remove excess dye. DiO‐labelled exosomes were incubated with DiI‐labelled cells for 24 hours and images of exosome uptake were obtained by fluorescent microscopy (Olympus).

### 2.5 Cell growth inhibition assay

The viability of NSCLC cells was determined by Cell Counting Kit (Dojindo) and detected at 490 nm with a microplate reader. Cells were seeded in DMEM at a density of 3 × 10^3^ in 96‐well plates overnight, then exposed to various concentrations of gefitinib for 72 hours. The supernatant was removed, and 100 μL DMEM containing 10% CCK‐8 solution was added to each well and incubated for 2 hours. All experiments were repeated in triple.

### 2.6 Western blot

Proteins were extracted with RIPA protein extraction reagent (Beyotime) containing 1% PMSF (Biotech Well), 1% protease inhibitor (Biotech Well) and 1% phosphatase inhibitor (Biotech Well). Approximately 20 μg of cell lysates were separated using 10% SDS‐PAGE and transferred onto nitrocellulose membranes (Pall), then incubated with specific antibodies diluted in TBST/5% skim milk powder at 4°C overnight and then washed with TBST for 3 times and incubated for 2 hours with horseradish peroxidase‐conjugated goat anti‐rabbit IgG (1:2000) (cell signalling technology) or goat antimouse IgG (1:2000) (Cell Signalling Technology) at room temperature. An enhanced chemiluminescent (Thermo Scientific) chromogenic substrate was used to visualize the bands. Antibodies for EGFR (1:2000), pEGFR (1:2000), ERK (1:2000), pERK (1:2000) and β‐actin (1:2000) were purchased from Cell Signalling Technology. Antibodies for AKT (1:2000) and pAKT (1:2000) were purchased from Epitomics (Burlingame).

### 2.7 In vitro wound‐healing assay

After cells reached 90% confluence in 6‐well plates, a linear wound was made by scraping the cell monolayer with a 200 μL pipette tip. After washing with PBS twice, the wound healing was carried out in serum‐free medium and photographed after 0 and 24 hours, and analysed by measuring the distance of migrating cells from five different areas for each wound.

### 2.8 Animal experiments

Animal experiments were undertaken in accordance with institutional guidelines, with the approval of our Institutional Animal Care and Use Committee. Four‐ to six‐week‐old male‐specific pathogen‐free (SPF) athymic BABL/c nude mice were used in our study. All mice were subcutaneous transplanted with PC9 cell in the right flanks (1 × 10^6^ PC9 cell in 100 μL PBS per mouse). After the tumour volumes reached 30‐50 mm^3^ (approximately 15 days), mice were randomly divided into 3 groups (control group, treatment with PC9‐derived exosome group and treatment with H1975‐derived exosome group). All groups received gefitinib (10 mg/kg) by oral gavage every day for 15 days. For the group treated with PC9 or H1975‐derived exosomes, exosomes (100 μg) were injected intratumorally every other day.

Tumour sizes were assessed every other day by a digital caliper and measured their length (*L*) and width (*W*). The tumour volumes were calculated using the formula: *V *= L * *W* ^2^/2. All the mice were killed by cervical dislocation for analysis after 15 days.

### 2.9 RNA isolation and qRT‐PCR analysis

We extracted total RNA from cells and exosomes by RNeasy Mini Kit (Qiagen). Real‐time PCR was performed on triplicate samples with miScript SYBR Green PCR Kit (Qiagen) and an MX3000P instrument. The miRNA levels were normalized against U6. All the primers were designed by Ruidi. The median of each triplicate set of values was used to calculate relative expression of miRNA by using 2^−ΔΔCt^ method. Sequences of primers used for qRT‐PCR in this study were listed as follows.

miR‐522‐3p: cgcgAAAATGGTTCCCTTTAGAGTGT
miR‐3648: GCC GCG GGG ATC GCC
miR‐1293: TGGGTGGTCTGGAGATTTGTGC
miR‐454‐3p: acgcgTAGTGCAATATTGCTTATAGGGT
miR‐874‐3p: CTGCCCTGGCCCGAG
miR‐500a‐5p: cgTAATCCTTGCTACCTGGGTGAGA
miR‐548f‐3p: gcgcgcgcgAAAAACTGTAATTACTTTT
miR‐576‐3p: agcgcgAAGATGTGGAAAAATTGGAATC
miR‐130a‐3p: acgCAGTGCAATGTTAAAAGGGCAT

### 2.10 Transfection experiment

Cells were seeded in 6‐well plate and incubated overnight. Transfection of miR‐522‐3p mimic (Ribobio) was performed using ribo*FECT* CP Reagent (Ribobio). Cells were harvested for downstream analysis after 48 hours of transfection.

### 2.11 Small RNA sequencing and bioinformatics

Exosomes were isolated from H1975 and PC9 cultures following the procedure described above. Before exosomal RNA extraction, exosomes were pretreated with RNase (Qiagen). Total RNAs were extracted from purified exosomes using RNeasy Mini Kit (Qiagen). The concentration and purity of small RNA in total RNAs were determined using the Qubit RNA Assay Kit in Qubit 2.0 Flurometer (Life Technologies) and NanoPhotometer spectrophotometer (IMPLEN). RNA integrity was evaluated by the RNA Nano 6000 Assay Kit of the Agilent Bioanalyzer 2100 System (Agilent Technologies). For the small RNA library, 3 μg RNA per sample was used as input material. We generated sequencing libraries by using NEBNext Multiplex Small RNA Library Prep Set for Illumina (NEB) and index codes were added to attribute sequences to each sample. Library quality was evaluated on the Agilent Bioanalyzer 2100 system by DNA High Sensitivity Chips.

Then, we performed the clustering of the index‐coded samples on a cBot Cluster Generation System using TruSeq SR Cluster Kit v3‐cBot‐HS (Illumia). Library preparations were sequenced on an Illumina Hiseq 2500/2000 platform and 50 bp single‐end reads were generated.

### 2.12 Immunocytochemistry and immunohistochemical (IHC) analysis

After seeded on 6 wells with glass coverslips, cells were fixed by paraformaldehyde for 15 minutes and treated with Triton X‐100 and blocked of the endogenous peroxidase by 3% hydrogen peroxidase. Then, we incubated cells with diluted primary antibodies (1:150) at 4°C overnight and incubated with HRP‐conjugated secondary antibody.

For IHC, paraffin‐embedded 5‐μm sections were deparaffinized and rehydrated. After primary and secondary antibody (see in Western blot) incubation, the slides were finally incubated with diaminobenzidine (DAB) (Dako) and counterstained with haematoxylin (Sigma Chemical Co).

### 2.13 Statistical analysis

All data analysis in our study was performed with GraphPad Prism (version 5.0, GraphPad Software, Inc). Differences between groups were analysed by Student's *t* test or analysis of variance (ANOVA). *P* < .05 was considered statistically significant.

## 3 RESULTS

### 3.1 Characterization of NSCLC cell‐derived exosomes

**Figure 1:**
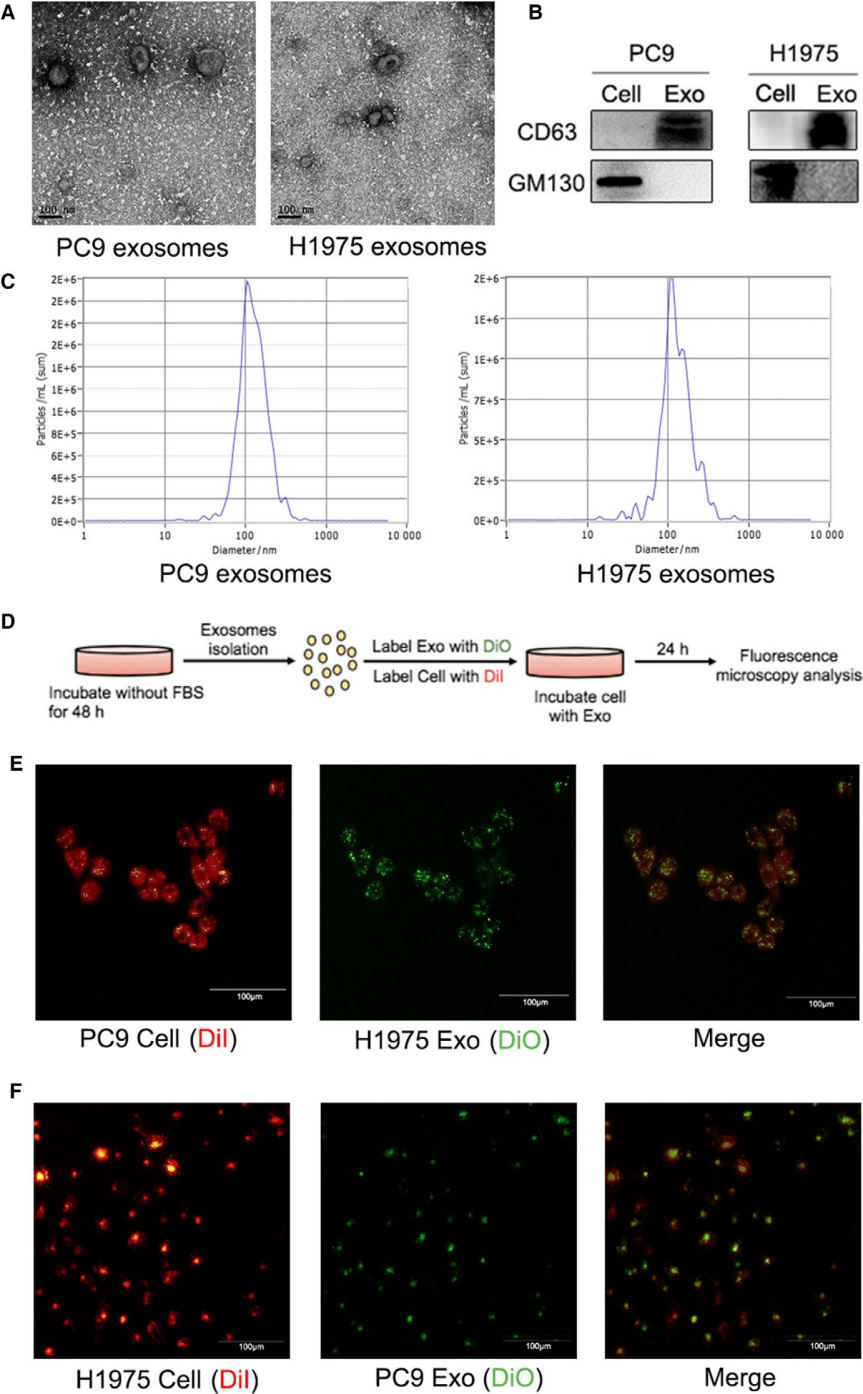
Characterization of exosomes derived from NSCLC cell lines and internalization of exosomes by NSCLC cells. A, Representative TEM images of exosomes isolated from the supernatants of NSCLC cells. Scale bar, 100 nm. B, NTA analysis of the size distribution and number of exosomes. C, Western blot analysis showed that exosomal marker CD63 was expressed in exosomes but not in cell lysate. In contrast, *cis*‐Golgi compartment‐specific marker GM130 was only detected in cell lysate but not in exosomes. D, The numbers of the exosomes derived from an equal number of cells were similar between the two cells by NTA analysis. E, Schematic diagram of the internalization of exosomes. F, Representative fluorescent and merge images about fluorescence microscopy observation of DiI‐labelled (red) PC9 cells 24 h after incubated with DiO‐labelled (green) exosomes. Scale bar, 100 μm. G, Representative fluorescent and merge images about fluorescence microscopy observation of DiI‐labelled (red) H1975 cells 24 h after incubated with DiO‐labelled (green) exosomes. Scale bar, 100 μm

To elucidate the effect of exosomes shed by NSCLC cells on gefitinib resistance, exosomes were derived from the supernatants of each cell type. We characterized and quantified exosomes by TEM, Western blot analysis and NTA. TEM revealed that the isolated exosomes were typical cup‐shaped membranous particles, approximately 100 nm in diameter (Figure 1A). NTA analysis showed that the size distribution for the two types of exosomes was 80‐150 nm (Figure 1B). Western blot analysis showed isolated exosomes were positive for exosomal marker CD63 but negative for the *cis*‐Golgi compartment‐specific marker GM130 (Figure 1C). Furthermore, the NTA analysis also demonstrated that the numbers of the exosomes derived from an equal number of cells were similar between the two cells (Figure 1D). Taken together, our results showed that the exosomes derived from H1975 cell and PC9 cell have no morphological, size or concentration differences.

### 3.2 NSCLC cell‐isolated exosomes can be uptaken and internalized by NSCLC cells

We next examined whether the exosomes isolated from PC9 and H1975 cell can be taken up and internalized by each other (Figure 1E). DiI‐labelled PC9 cells (Figure 1F) or H1975 cells (Figure 1G) were incubated with DiO‐labelled H1975 or PC9 cell‐derived exosomes for 24 hours, and exosome uptake by recipient cells was observed by fluorescence microscopy. These results indicated that NSCLC cell‐isolated exosomes can be uptaken and internalized by NSCLC cells, and NSCLC cells can communicate with each other through exosomes.

### 3.3 H1975 cell‐derived exosomes induce gefitinib resistance in PC9 cell in vitro

**Figure 2:**
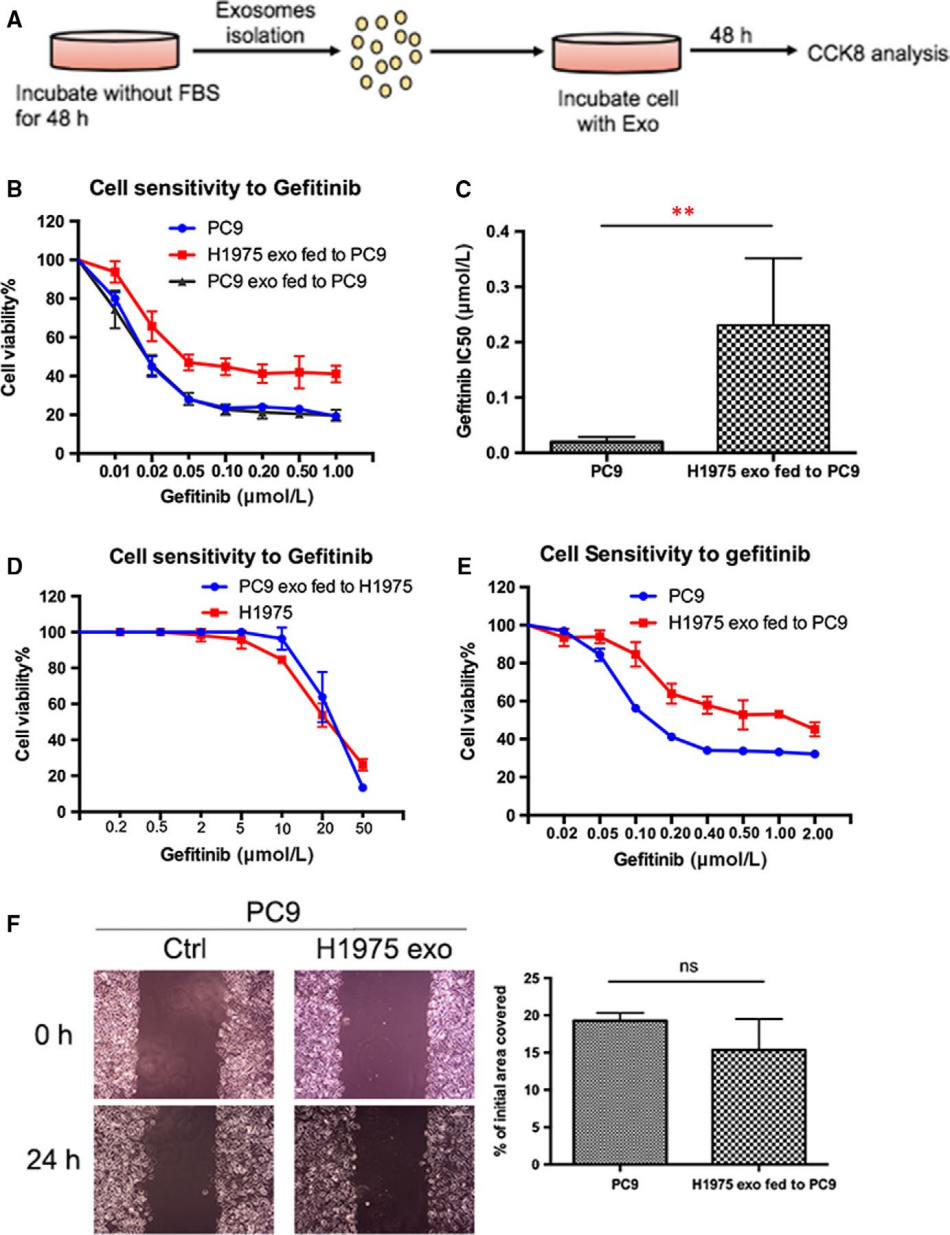
H1975 cell‐derived exosomes induce gefitinib resistance in PC9 cell in vitro. A, Schematic diagram of CCK8 analysis. B, CCK‐8 assays of PC9 cell incubated with H1975‐derived exosomes or PC9‐derived exosomes or PBS for 48 h followed by gefitinib treatment at indicated concentrations for 72 h. H1975‐derived exosomes could induce gefitinib resistance in PC9 cell, and exosomes released by PC9 cell do not have this effect. C, IC_50_ values of PC9 cell incubated with H1975‐derived exosomes was significantly elevated than that of PC9‐derived exosomes. *P < .05, **P < .01. D, CCK‐8 assays of H1975 cell incubated with PC9‐derived exosomes for 48 h and treated with gefitinib at indicated concentrations for 72 h. Incubation of exosomes derived from PC9 with H1975 could not transfer gefitinib sensitivity to H1975 cell. E, PC9 cells incubated with H1975‐derived exosomes for 48 h then replaced with fresh culture medium for 3 d and CCK‐8 assays were conducted. F, Wound‐healing assays of PC9 cell incubated with indicated exosomes. The addition of exosomes released by H1975 cells did not result in a change in the capacity of migratory

Since growing evidence indicated that exosomes derived from drug‐resistant cells can transfer drug resistance to sensitive cells,^13^ we next investigated whether H1975 cell‐isolated exosomes can induce gefitinib resistance in PC9 cell in vitro. We incubated PC9 cell with H1975 or PC9 cell‐derived exosomes for 48 hours and determined the half maximal inhibitory concentration (IC_50_) of gefitinib in PC9 cell by using CCK‐8 assays (Figure 2A). Our results suggested that incubation with exosomes isolated from H1975 could induce gefitinib resistance in PC9 cell, the IC_50_ was significantly higher than incubation with PC9‐derived exosome or control group (*P* < .01). However, incubation with exosomes derived from PC9 cell do not have this effect (Figure 2B,C). Meanwhile, we found that the incubation of exosomes derived from PC9 with H1975 cell could not transfer gefitinib sensitivity to H1975 cell (Figure 2D). In addition, we noticed that the exosome‐conferred resistance in PC9 cell sustained for at least 3 days after the removal of resistance exosomes (Figure 2E). Moreover, wound‐healing assays showed that the addition of exosomes released by H1975 cells did not result in a change in the capacity of migratory (Figure 2F). Together, these data indicated that H1975 cell‐isolated exosomes can transfer gefitinib resistance in PC9 cell in vitro*.*

### 3.4 H1975 cell‐derived exosomes induce gefitinib resistance in PC9 cell in vivo

**Figure 3:**
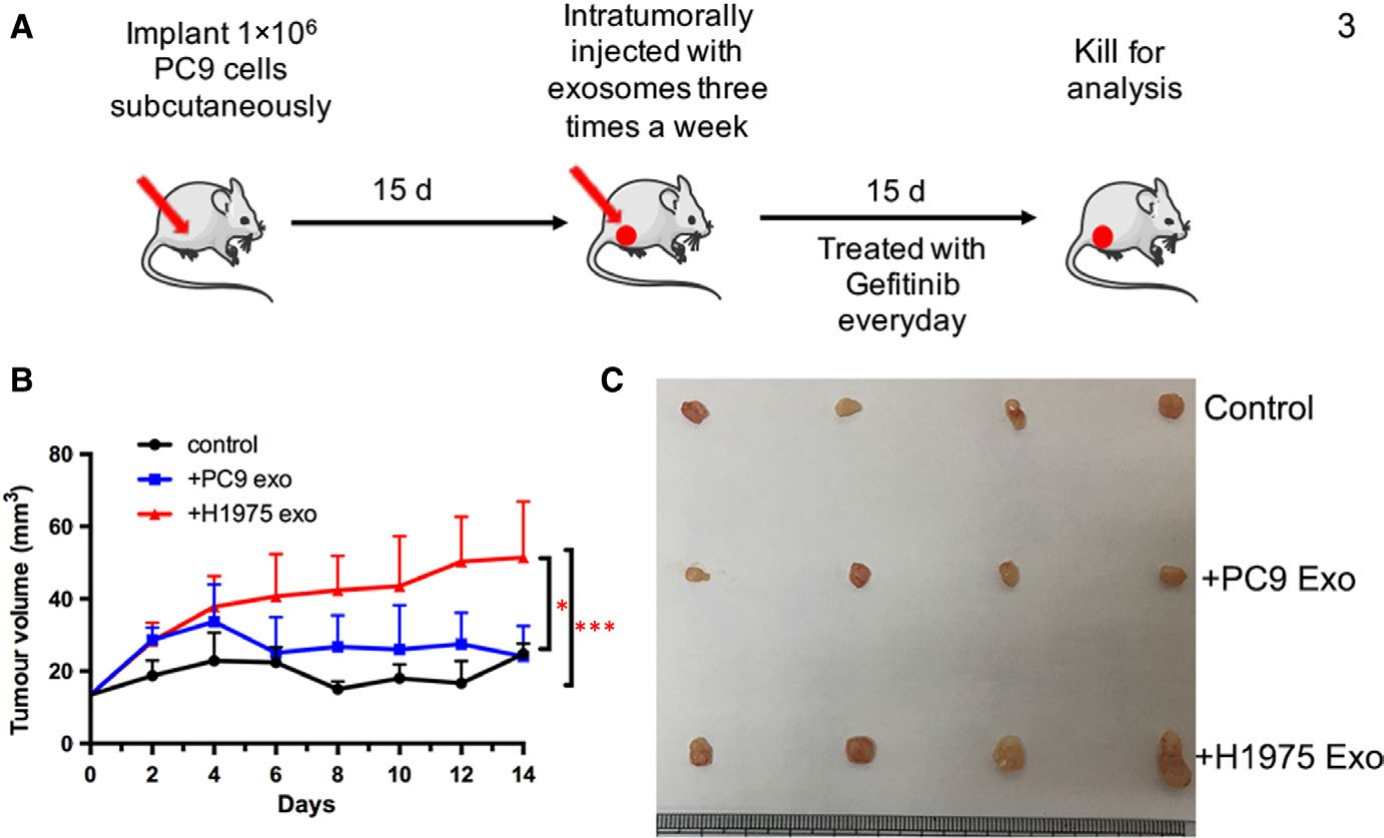
H1975 cell‐derived exosomes induce gefitinib resistance in PC9 cell in vivo. A, Schematic diagram of in vivo animal experiments. B, C, Subcutaneous xenograft analysis of PC9 cells (1 × 10^6^ cells) in nude mice with intratumoral injection of indicated exosomes upon gefitinib (10 mg/kg) treatment. Tumour growth curves in mice were shown. The volumes of tumour treated with gefitinib plus with H1975‐derived exosomes were significantly larger than those treated with gefitinib alone or together with PC9‐derived exosomes. *P < .05, **P < .01

Next, we established a subcutaneous transplantation tumour model in athymic nude mice to examine whether H1975 cell‐isolated exosomes can promote gefitinib resistance in PC9 cell in vivo. When the tumour volume reached 30‐50 mm^3^, mice were treated with gefitinib every day together with PC9‐ or H1975‐derived exosomes or PBS intratumoral injection three times a week (Figure 3A). We observed that the tumours of mice treated with gefitinib plus with H1975‐derived exosomes were significantly larger than those of mice treated with gefitinib alone or together with PC9‐derived exosomes (*P* < .05). Meanwhile, the tumour volumes had no significant difference between intratumoral injection with PC9‐derived exosome group and control group, as shown in Figure 3B,C. These results indicated that exosomes released by EGFR‐TKI–resistant cell H1975 can inhibit the therapeutic effects of gefitinib and promote tumour growth in vivo.

### 3.5 H1975 cell‐derived exosomes promote gefitinib resistance via PI3K/AKT pathway

**Figure 4:**
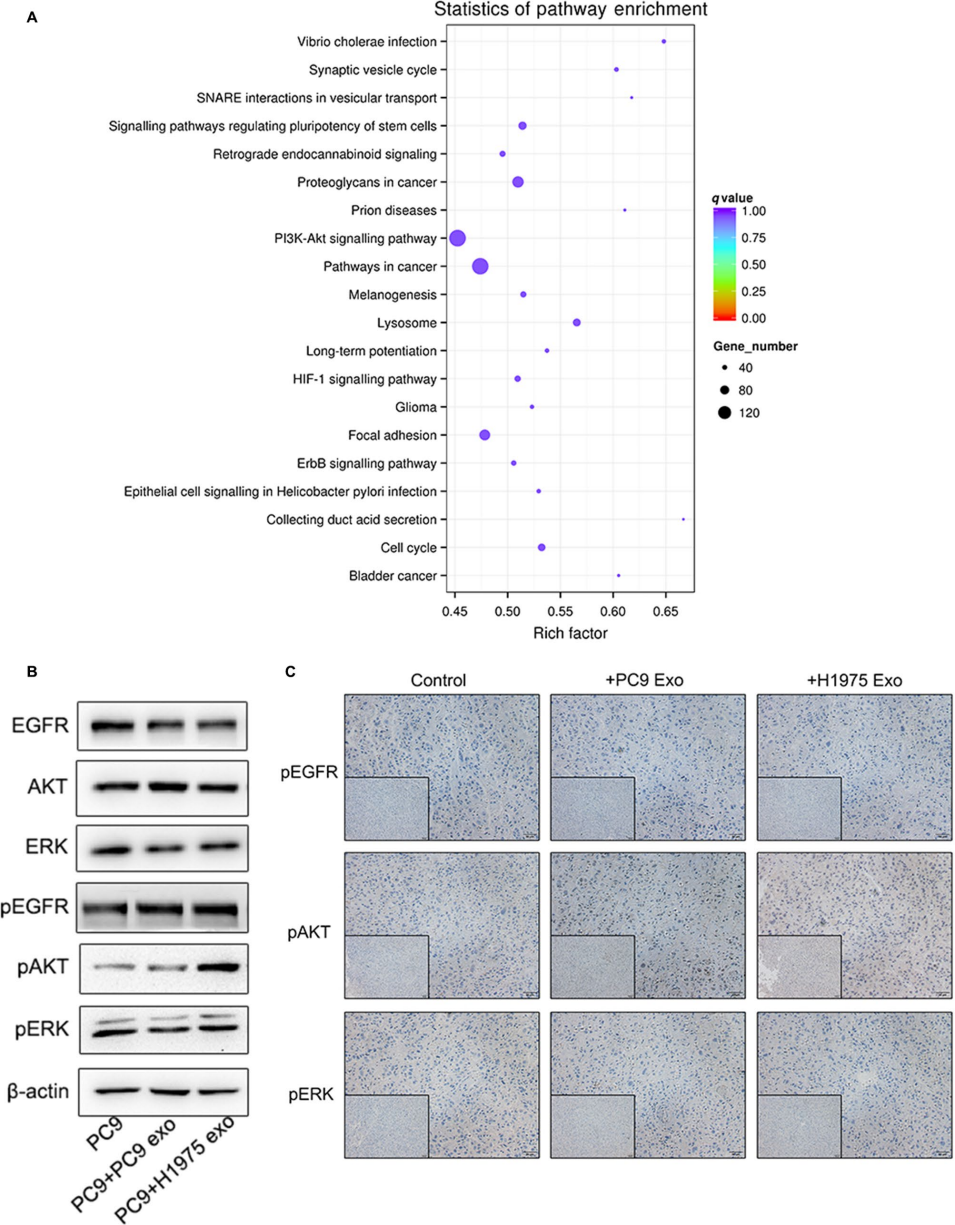
H1975 cell‐derived exosomes promote gefitinib resistance via PI3K/AKT pathway. A, Signalling pathways of differential miRNA target gene enrichment analysis. B, Western blot analysis about the proteins of EGFR and its downstream proteins in PC9 cell incubated with H1975 cell‐derived exosomes, PC9 cell‐derived exosomes and PBS for 48 h. C, Immunohistochemical analysis about the expression level of pEGFR, pAKT, pERK in the group treated with H1975‐derived exosomes, treated with PC9‐derived exosome and the control groups

To explore the potential mechanisms, we isolated and purified RNA from the exosomes released from PC9 and H1975 cell for small RNA sequencing. According to the latest KEGG (Kyoto Encyclopedia of Genes and Genomes) database, results of the differential miRNA target gene enrichment analysis showed that PI3K/AKT signalling pathway had the highest degree of enrichment and suggested a role in transferring gefitinib resistance (Figure 4A). Western blot analysis showed that in vitro the treatment with H1975 cell‐derived exosomes for 48 hours increased the expression of phosphor‐AKT (pAKT) in PC9 cell compared to the treatment with PC9 cell‐derived exosome or control group (Figure 4B), while the expressions of total EGFR, total AKT, total ERK, phosphor‐EGFR (pEGFR) and phosphor‐ERK (pERK) were not changed.

Meanwhile, we also used immunohistochemical analysis to confirm that exosomes derived from H1975 cell induce gefitinib resistance via PI3K/AKT signalling pathway in vivo*.* Our results revealed that the expression level of pAKT in the group treated with H1975‐isolated exosomes was significantly higher than that in the group treated with PC9‐derived exosome or control group. Therefore, these in vivo data complemented the in vitro experiments.

We further examined the expression of EMT markers to assess whether H1975 cell‐derived exosomes regulate gefitinib resistance through EMT. Immunocytochemistry revealed that the treatment of H1975 did not lead to increased expression of vimentin or decreased expression of E‐cadherin (Figure S1). These findings ruled out the possibility that H1975 cell‐derived exosomes regulate gefitinib resistance through EMT.

Taken together, our results indicated that exosomes derived from EGFR‐TKI–resistant cell H1975 could induce gefitinib resistance in sensitive cell PC9 through activating PI3K/AKT signalling pathway.

### 3.6 Differentially expressed miRNAs in exosomes

**Figure 5:**
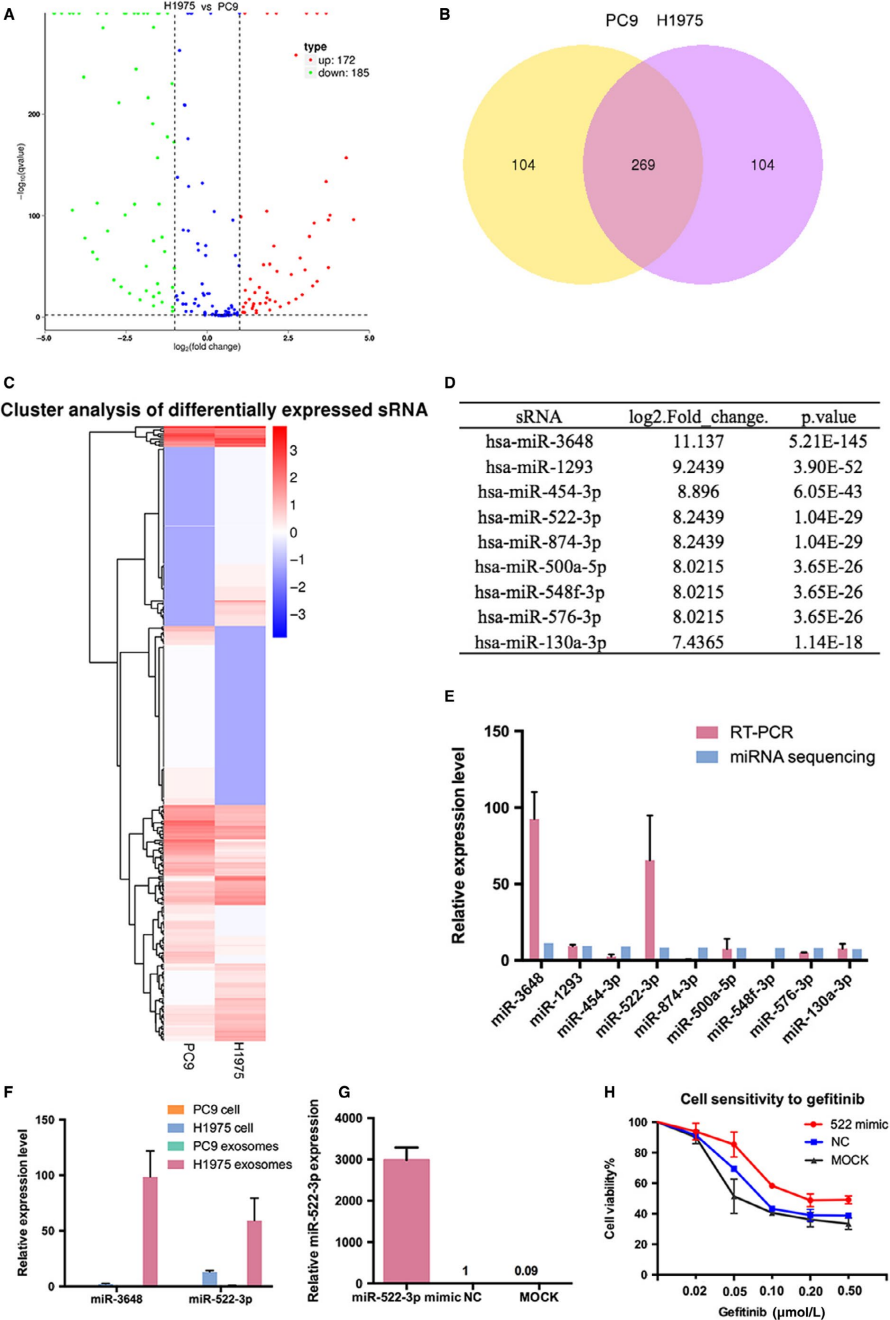
Differentially expressed miRNAs in the exosomes. A, Volcano plots about the differentially expressed miRNAs in exosomes derived from H1975 and PC9 cells. B, Differential miRNA Wayn Diagram of the distribution of differentially expressed miRNAs in exosomes. C, The expressions of small RNAs in exosomes isolated from PC9 and H1975 cell are presented in a heat map. D, E, The expression levels of the most significantly highly expressed in exosomes derived from H1975 cell and validated by qRT‐PCR. MiR‐3648 and miR‐522‐3p were the two most differentially expressed miRNAs. F, The expression levels of miR‐3648 and miR‐522‐3p in PC9 cell, H1975 cell, exosomes derived from PC9 cell and H1975 cell. G, The expression level of miR‐522‐3p in PC9 cell by the transfection of miR‐522‐3p mimic was significantly higher than transfected with miR‐522‐3p control. H, CCK‐8 assays of PC9 cell transfected with miR‐522‐3p mimic. Up‐regulation of miR‐522‐3p increased gefitinib resistance in PC9 cell

Small RNA sequencing was used to compare the small RNA expression profiles between exosomes derived from PC9 and H1975 cell. The expression levels of miRNA were estimated by TPM (transcript per million) through the following criteria.^19^ Differential expression analysis of the two samples was performed using the DEGseq R package.^20^ Volcano plots was utilized to infer the overall distribution of differential miRNAs. Results indicated that compared with the exosomes shed by PC9 cell, 172 differentially expressed miRNAs were significantly up‐regulated and 185 differentially expressed miRNAs were significantly down‐regulated in the exosomes released from H1975 cell (Figure 5A). Differential miRNA Wayn Diagram demonstrated the distribution of differentially expressed miRNAs in PC9‐derived exosomes and H1975‐derived exosomes (Figure 5B). The heat map demonstrated the relative expression of small RNAs in exosomes shed by PC9 and H1975 cells and showed a clear distinction between the two groups (Figure 5C).

### 3.7 MiR‐522‐3p up‐regulation increased gefitinib resistance in PC9 cell

Among the miRNAs identified in exosomes, we examined the first few miRNAs which were significantly highly expressed in exosomes shed by H1975 cell (Figure 5D) and validated the expression level by qRT‐PCR. The validation results indicated that miR‐3648 and miR‐522‐3p were the two most differentially expressed miRNAs (Figure 5E). Hence, we used qRT‐PCR to validate the expressions of miR‐3648 and miR‐522‐3p in PC9 cell, H1975 cell, exosomes derived from PC9 cell and H1975 cell. The findings illustrated that the expression level of miR‐522‐3p was significantly higher in H1975 cell and exosomes derived from H1975 cell than PC9 cell and exosomes derived from PC9 cell (Figure 5F). Previous study has shown that inhibition of miR‐522 can suppress proliferation of NSCLC cells and induce apoptosis.^21^ Therefore, we up‐regulated the expression level of miR‐522‐3p in PC9 cell by transfection of miR‐522‐3p mimic. As shown in Figure 5G, the relative expression of miR‐522‐3p of PC9 cell transfected with miR‐522‐3p mimic was significantly higher than transfected with miR‐522‐3p control. CCK8 assay showed that up‐regulation of miR‐522‐3p increased gefitinib resistance in PC9 cell (Figure 5H). The results suggested that miR‐522‐3p may be a possible mechanism of the transmission of EGFR‐TKI resistance.

## 4 DISCUSSION

The discovery and application of EGFR‐TKIs has heralded the beginning of molecularly targeted therapy for NSCLC. However, acquired resistance is a major obstacle to the failure of targeted therapy. Drug resistance is a multifaceted problem, although the mechanisms of acquired resistance of *EGFR*‐mutant NSCLC to EGFR‐TKIs have been identified,^4^ little is known about the transmission of drug resistance between EGFR‐TKI–resistant cells and sensitive cells and this needs to be further elucidated. Recent researchers have reported that small populations of drug‐resistant cells in patients continue to share molecular characteristics of the drug‐resistant cells, contributing to a attenuated apoptotic response to subsequent therapies.^7^ Therefore, we put our sights into the transmission of drug resistance between lung cancer cells.

Exosomes were first discovered as a mechanism to eliminate unwanted components from a cell.^22^ Recently, emerging evidence has begun to describe the roles of exosomes in tumour progression and aggressiveness.^10^, ^23^ Exosome transport between cell populations in the extracellular environment is considered to be an effective means of modulating cell signalling and biological function in recipient cells.^24^ Since exosomes are now regarding as emerging mediators of cell‐to‐cell communication, studying their relationship to drug resistance is becoming an exciting area. Previously researchers have suggested that exosomes derived from drug‐resistant cells leading to the spread of drug resistance by transferring their cargo to drug‐sensitive cells.^13^ This phenomenon has been described in several other tumour types, like renal cancer and breast cancer,^14^, ^25^ which suggest that in lung cancer, drug resistance may also be transmitted via exosomes. In previous researches, Feng et al^15^ demonstrated that exosome‐transmitted miR‐222‐3p was shown as a principle regulator of gemcitabine resistance and malignant characteristics in NSCLC. Qin et al^26^ proved that DDP‐resistant lung cancer cells confer recipient cells' resistance to DDP in an exosomal‐miR‐100‐5p–dependent manner. The discovery of this mechanism of drug–resistant transmission has been highlighted and provided as a new opportunity not only to find means to counteract this phenomenon, but also to identify biomarkers of drug resistance.^14^, ^15^ Therefore, we first aimed to investigate the roles of exosomes isolated from EGFR‐TKI–resistant NSCLC cells carrying *T790M* mutation to transferring drug resistance to sensitive cells and explored the potential mechanisms. In our experiment, EGFR‐TKI–sensitive cell PC9 and resistant cell harbouring *T790M* mutation H1975 were used. We found that exosomes released by EGFR‐TKI–resistant cell H1975 which harbouring *T790M* mutation can promote gefitinib resistance in EGFR‐TKI–sensitive cell PC9 both in vitro and in vivo*.* These results were consistent with our previous assumptions, raising the possibility that intercellular transfer of exosomes may be a novel mechanism for the transfer of EGFR‐TKI resistance.

Valadi et al^27^ first found the transfer of miRNA by exosomes and presented a view that this may be an important mechanism for genetic exchange between cells. This has generated interest in exploring the functions of miRNAs shuttled by exosomes in drug transmission. In previous researches, exosomes have been demonstrated to shuttle nucleic acids from a donor to receipt cells.^27^, ^28^ In breast cancer, studies have shown that exosomes shed by drug‐resistant breast cancer cells could transmit resistance to sensitive cells, such effect may be partly attributed to the intercellular transfer of miR‐222.^25^, ^29^ In NSCLC, exosome‐transmitted miR‐222‐3p also functioned as a principle regulator of gemcitabine resistance and exosome‐transmitted miR‐100‐5p regulates DDP resistance.^15^, ^26^ In addition to miRNA, Le Qu et al^14^ also identified that exosome‐mediated delivery of lncARSR could offer sunitinib resistance to sensitive cells in renal cancer. To elucidate the functions of exosomal miRNA in EGFR‐TKI resistance, a small RNA sequencing was performed to identify differentially expressed miRNAs between the two cells. The expression level of miR‐522‐3p was significantly higher in H1975 cell and exosomes derived from H1975 cell than PC9 cell and exosomes derived from PC9 cell by RT‐PCR validation. Up‐regulation of miR‐522‐3p by miR‐522‐3p mimics increased gefitinib resistance in PC9 cell. The results indicated that miR‐522‐3p may be a possible mechanism in the transmission of EGFR‐TKI resistance.

Besides, epithelial‐mesenchymal transition (EMT) has been associated with acquired resistance to EGFR‐TKIs, with a decrease in epithelial markers such as E‐cadherin and an increase in mesenchymal markers such as vimentin.^30^ Richard et al^31^ also found that mesenchymal NSCLC‐derived exosomes from chemoresistant cells are capable of transferring resistance to chemosensitive epithelial cells. Therefore, we explored whether EMT also plays a role in the transmission of EGFR‐TKI resistance. Results of wound‐healing assay ruled out the possibility that H1975 cell‐derived exosomes regulate gefitinib resistance through EMT.

PI3K/AKT is a key downstream signalling pathway of EGFR and abnormal activation of the PI3K/AKT signalling pathway is one of the most relevant mechanisms of acquired targeted therapies resistance in EGFR‐mutated NSCLC patients.^32^, ^33^ Our results demonstrated that EGFR‐TKI–resistant cell H1975–derived exosomes can induce gefitinib resistance in sensitive cell PC9 through activating PI3K/AKT signalling pathway. Besides, based on the latest KEGG database, results of the differential miRNA target gene enrichment analysis indicated that PI3K/AKT signalling pathway had the highest degree of enrichment and suggested a role in transferring gefitinib resistance.

We first discussed the roles of exosomes shed by EGFR‐TKI–resistant NSCLC cells harbouring *T790M* mutation in drug resistance transmission. However, we only used two cells, which is a major limitation in our study. Further investigations in more cell cultures are needed to reinforce our results. Our results also need to be confirmed in patients. Moreover, with the discovery and application of third‐generation EGFR‐TKIs, like osimertinib,^34^ research about third‐generation EGFR‐TKI resistance is underway, whether exosomes also participate in the transfer of third‐generation EGFR‐TKI resistance also needs to be elucidated. Further investigations are warranted.

Lung cancer is a heterogeneous disease and intratumoural heterogeneous becomes more complicated after targeted therapy. Tumour heterogeneous and drug resistance have been demonstrated to be important in cancer therapy.^35^, ^36^ Researchers have reported that genetic resistance may come from small groups of drug‐resistant cells in patients. These resistant clones may continue to share the molecular characteristics of the drug‐resistant cells, contributing to a attenuated apoptotic response to subsequent therapies.^7^ The dynamic interplay between tumour cells is an important event that contributes to drug resistance. And the microenvironment signals are provided by exosomes, which play a key role in tumour host crosstalk. Our findings first showed the roles of exosomes in EGFR‐TKI resistance transmission and provided novel insights into further understanding the contribution of tumour cell plasticity to drug resistance transfer.

Consistent with the view that exosomes are key participants in intercellular communication, our findings demonstrated that exosome‐transmitted miR‐522‐3p shed by EGFR‐TKI–resistant cells carrying *T790M* mutation can induce gefitinib resistance in sensitive cell via activating PI3K/AKT signalling pathway both in vitro and in vivo. And the results revealed a novel mechanism of acquired resistance to EGFR‐TKIs in NSCLC.

## CONFLICT OF INTEREST

All the authors declare that they have no competing interests.

## **AUTHORS**'** CONTRIBUTIONS**

Xiaozhen Liu, Tao Jiang, Xuefei Li, Chao Zhao, Jiayu Li, Fei Zhou, Limin Zhang, Sha Zhao, Yijun Jia, Jinpeng Shi, Guanghui Gao, Wei Li, Xiaoxia Chen, Jing Zhao, Chunxia Su, Shengxiang Ren, Caicun Zhou, X.Z Liu, T Jiang and C.C Zhou designed this study. X.Z Liu, T Jiang, L.M Zhang, S.Z, Y.J Jia and J.P Shi carried out experiments. X.F Li and C.Z provided experimental methods. J.Y Li, F Zhou, G.H Gao, W Li, X.X Chen, J Zhao, C.X Su and S.X Ren took on the statistical analysis. X.Z Liu, T Jiang and F Zhou drafted the manuscript. C.X Su, S.X Ren and C.C Zhou provided critical suggestions and revised the manuscript. All the authors had the approval of the submitted and published versions.

## Data Availability

The data used to support findings of the study are available from the corresponding author upon request.
