# Supplementary figures and images for: Exosomes transmit T790M mutation‐induced resistance in EGFR‐mutant NSCLC by activating PI3K/AKT signalling pathway
Source: J Cell Mol Med. 2020 Jan 2;24(2):1529–40. doi: 10.1111/jcmm.14838 (PMC6991626; doi:10.1111/jcmm.14838)

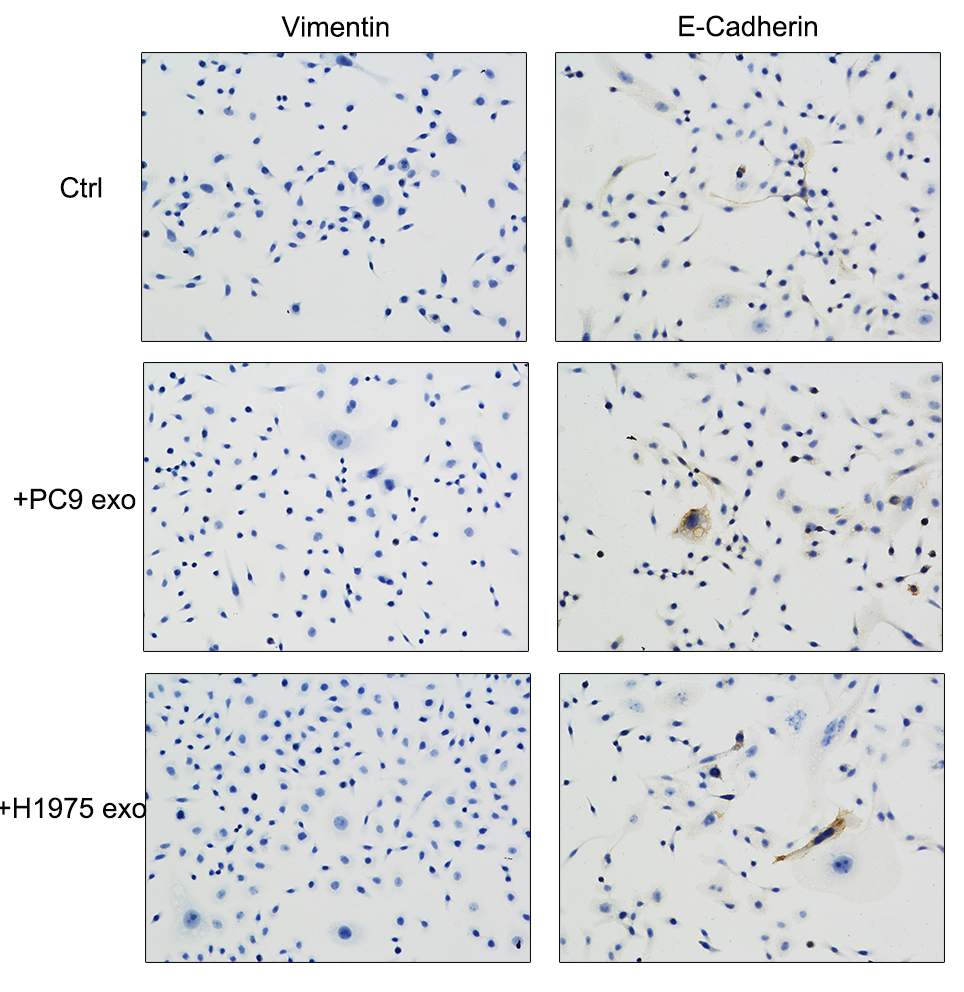

Supplement: Supplementary file 1 [file JCMM-24-1529-s001.tif]
